# Large language models for efficient whole-organ MRI score-based reports and categorization in knee osteoarthritis

**DOI:** 10.1186/s13244-025-01976-w

**Published:** 2025-05-14

**Authors:** Yuxue Xie, Zhonghua Hu, Hongyue Tao, Yiwen Hu, Haoyu Liang, Xinmin Lu, Lei Wang, Xiangwen Li, Shuang Chen

**Affiliations:** 1 Department of Radiology & Institute of Medical Functional and Molecular Imaging, Huashan Hospital, Fudan University, Shanghai, China; 2 Digital & Automation, Siemens Shanghai Medical Equipment Ltd., Shanghai, China

**Keywords:** Large language model, Osteoarthritis, Chain-of-thought, MRI

## Abstract

**Objectives:**

To evaluate the performance of large language models (LLMs) in automatically generating whole-organ MRI score (WORMS)-based structured MRI reports and predicting osteoarthritis (OA) severity for the knee.

**Methods:**

A total of 160 consecutive patients suspected of OA were included. Knee MRI reports were reviewed by three radiologists to establish the WORMS reference standard for 39 key features. GPT-4o and GPT-4o-mini were prompted using in-context knowledge (ICK) and chain-of-thought (COT) to generate WORMS-based structured reports from original reports and to automatically predict the OA severity. Four Orthopedic surgeons reviewed original and LLM-generated reports to conduct pairwise preference and difficulty tests, and their review times were recorded.

**Results:**

GPT-4o demonstrated perfect performance in extracting the laterality of the knee (accuracy = 100%). GPT-4o outperformed GPT-4o mini in generating WORMS reports (Accuracy: 93.9% vs 76.2%, respectively). GPT-4o achieved higher recall (87.3% s 46.7%, *p* < 0.001), while maintaining higher precision compared to GPT-4o mini (94.2% vs 71.2%, *p* < 0.001). For predicting OA severity, GPT-4o outperformed GPT-4o mini across all prompt strategies (best accuracy: 98.1% vs 68.7%). Surgeons found it easier to extract information and gave more preference to LLM-generated reports over the original reports (both *p* < 0.001) while spending less time on each report (51.27 ± 9.41 vs 87.42 ± 20.26 s, *p* < 0.001).

**Conclusion:**

GPT-4o generated expert multi-feature, WORMS-based reports from original free-text knee MRI reports. GPT-4o with COT achieved high accuracy in categorizing OA severity. Surgeons reported greater preference and higher efficiency when using LLM-generated reports.

**Critical relevance statement:**

The perfect performance of generating WORMS-based reports and the high efficiency and ease of use suggest that integrating LLMs into clinical workflows could greatly enhance productivity and alleviate the documentation burden faced by clinicians in knee OA.

**Key Points:**

- GPT-4o successfully generated WORMS-based knee MRI reports.
- GPT-4o with COT prompting achieved impressive accuracy in categorizing knee OA severity.
- Greater preference and higher efficiency were reported for LLM-generated reports.

**Graphical Abstract:**

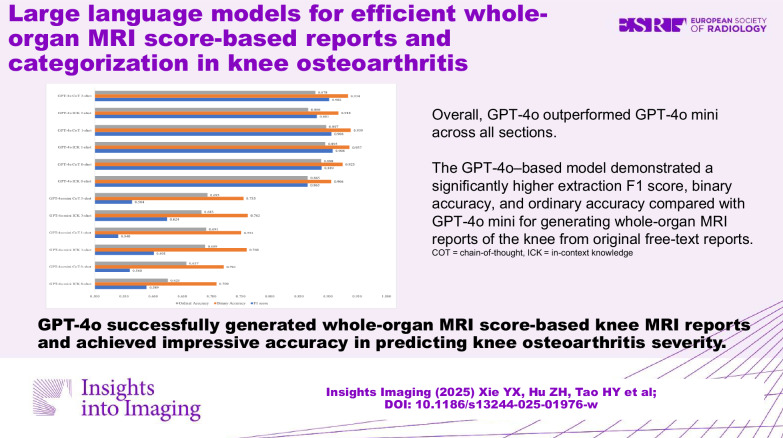

## Introduction

Knee osteoarthritis (OA) remains the most common cause of knee pain and functional impairment, particularly among the elderly [1, 2]. Clinically, knee OA is characterized by the gradual deterioration of articular cartilage at the ends of bones and all of the other structural changes in joint tissues [3]. Without appropriate intervention, these structural changes can increase the risk of dysfunction and may eventually necessitate knee replacement surgery. As such, early diagnosis and accurate classification of severity are crucial for effective management in clinical practice. MRI has significantly advanced our understanding of the morphologic features that contribute to the development of knee OA. The Whole-organ MRI score (WORMS) [3] is the most commonly used MRI classification system in clinical research for assessing the severity of knee abnormalities in OA [4–6]. However, despite its widespread use in research, it is very time-consuming in clinical settings due to the large number of features involved. Furthermore, the extensive details accumulated within free-text reports can pose challenges for clinicians and surgeons to interpret, increasing the risk that key findings may be overlooked or misinterpreted, especially in tense clinical environments [7]. These challenges underscore the need for reliable automatic tools that can summarize free-text radiology reports into structured WORMS-based reports, which could greatly benefit the management of knee OA.

Large language models (LLMs) have recently revolutionized natural language processing, demonstrating robust performance on traditional tasks with minimal examples (few-shot learning) [8, 9]. They have also shown the ability to reason at higher levels of complexity and apply knowledge in specialized domains such as Radiology. Several exciting potential applications of LLMs in Radiology have highlighted their potential to improve patient care [10–12]. Among them, only one study has focused specifically on knee MRI. This research compared the performance of several LLM models in report summarization and showed approximately 70% accuracy in fluency and consistency with reports generated by radiologists [13]. The encouraging results of this feasibility study suggest that LLMs have the potential to provide a preliminary summarization of free-text reports into structured reports for knee MRI.

In this study, it was hypothesized that LLMs could efficiently generate whole-organ, multi-feature knee MRI reports and accurately predict OA severities. Hence, the purpose of this study was to evaluate the performance of LLMs in automatically generating WORMS-based structural reports from original free-text reports and to explore their ability to classify knee OA severity using multiple prompting strategies.

## Methods

This retrospective study was performed at an academic hospital, with institutional review board approval and the requirement of informed consent waived due to the use of retrospective nonidentifiable data.

### Report collection and preparation

**Fig. 1:**
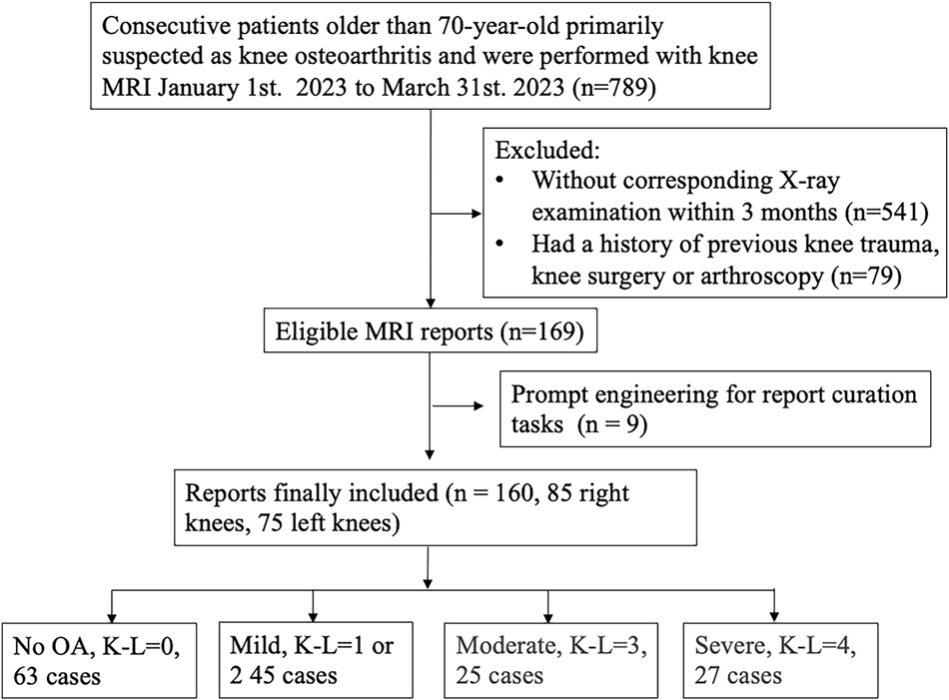
Study flowchart. The OA severity was categorized according to the K–L classification. OA, osteoarthritis

All consecutive patients aged 70 years and older, who presented with knee pain for at least three months and were primarily suspected of having knee OA in the outpatient setting with knee MRI examination from January 1 to March 31, 2023, were reviewed. Patients without corresponding X-ray examinations within three months at our institution were excluded (Fig. 1). All personal health information and potentially identifiable data were manually removed from each report following the Health Insurance Portability and Accountability Act Safe Harbor method [14].

### Reference standard of features and OA categorization

All original MRIs were reviewed by three radiologists to establish the reference standard and ordinary classification of thirty-nine features (thirty-eight whole-organ features according to the WORMS system, along with the laterality). Corresponding X-rays were interpreted to establish OA severity reference based on the Kellgren–Lawrence (K–L) classification. Specifically, the reports were independently reviewed by two radiologists (with 16 years and 13 years of experience, respectively), with disagreements discussed with a third radiologist to reach a consensus (with 39 years of experience). Additionally, a binary indicator of abnormality for each feature was derived by recoding the ordinal score of 0 for negative (normality) and scores of greater than 0 for positive (abnormality). Detailed classification criteria for each feature and OA criteria are provided in Appendix E1.

### LLMs and prompts used

In this research, OpenAI’s mainstream GPT-4o and GPT-4o-mini models were employed to generate structured reports without any specific training, as reported previously [10, 15, 16]. The latest versions of gpt-4o-2024-08-06 and gpt-40-mini-2024-07-18 were accessed via OpenAI API 1.50.2. First, the WORMS grading criteria were incorporated into the prompts. Then, the original MRI reports were input into the models, and a structured output in JSON format was configured using the “response_format” feature of the API. Next, if the COT method was used, step-by-step rules were provided to guide the extraction of relevant information. Subsequently, corresponding examples were added based on the number of shots. Finally, Python scripts were executed to obtain the WROM grading results for each feature. The temperature was set to zero to minimize the variability of the LLMs’ outputs.

The classification of individual features, as well as the prediction of OA severity, was explored based on different prompt strategies. Specifically, two prompting strategies, in-context knowledge (ICK) and chain-of-thought (COT), were used, with 0-shot, 1-shot, and 3-shot learning approaches tested for each. All six strategies were applied to both models (GPT-4o and GPT-4o mini), resulting in a total of twelve model configurations.

### Performance evaluations for the LLM models

**Fig. 2:**
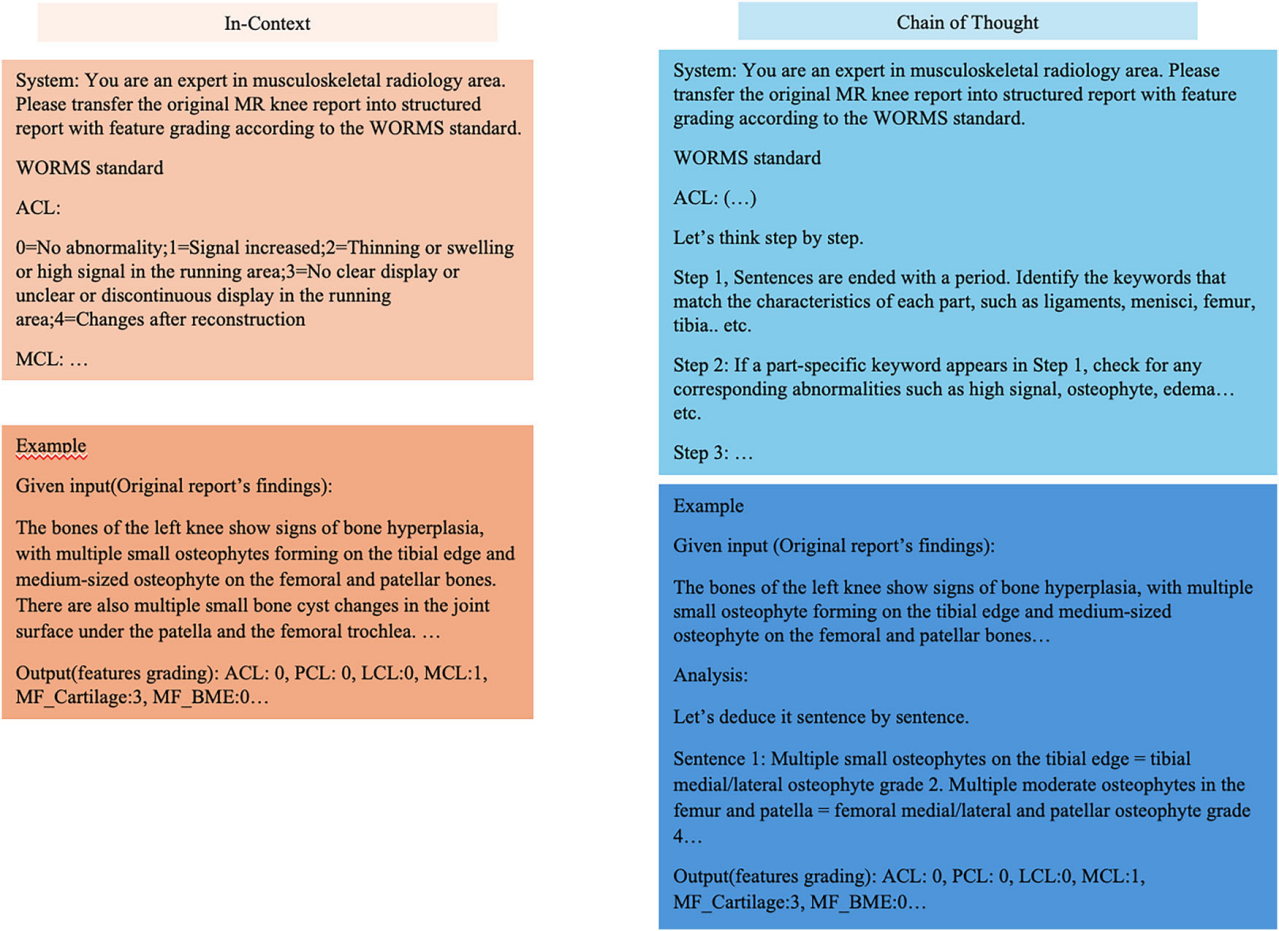
The diagram shows prompting strategies for models tasked with extracting and classification of individual features to create whole-organ MRI reports from original free-text reports. The provided template included whole-organ features and corresponding classification criteria for the knee MRI. Identical prompts were used for both GPT-4o and GPT-4o mini. Generated reports were simultaneously used for categorizing OA severity. ACL, anterior cruciate ligament; MCL, medial collateral ligament; PCL, posterior collateral ligament; LCL, lateral collateral ligament; MF, medial femoral; BME, bone marrow edema

Performance matrices (recall, precision, accuracy, and F1 scores) for automatically generating WORMS-based MRI reports, classifying individual features, and predicting OA severity were calculated by comparing them with the reference standard. Additionally, accuracy was calculated using both ordinal and binary classification methods. Figure 2 illustrates the prompting strategy used to guide the model in extracting and classifying individual features and generating WORMS-based knee reports from original free-text reports by the LLMs. Four orthopedic surgeons (with 33 years, 18 years, 17 years, and 12 years of experience, respectively) were consulted to determine an acceptable accuracy threshold for an OA severity prediction tool in real-life practice. The surgeons indicated that, to be useful for initial review, the tool must exceed an average accuracy of 85% (correct categorizations/total number of cases) based on binary classification and 75% based on ordinal classification [10].

### Orthopedic surgeon review

To assess the quality of the LLM-generated WORMS-based reports, four orthopedic surgeons independently annotated each report across several specific dimensions through pairwise comparisons. A 5-point Likert scale was utilized for the evaluation, covering the surgeons’ preference for each report and the difficulty of extracting key information; higher scores indicated less preference or greater difficulty (Table 1). Additionally, the time spent by the surgeons on each report, measured in seconds, was recorded.

**Table 1:** Likert scales for the orthopedic surgeon review

Item	1	2	3	4	5
Preference	Very like	Like	Medium	Dislike	Very dislike
Difficulty in extracting key information	Very easy	Easy	Medium	Difficulty	Very difficult

A 5-point Likert scale was utilized for the evaluation, with higher scores indicating less preference or greater difficulty in extracting key information

### Statistical analysis

Performance between GPT-4o and GPT-4o mini models was calculated as recall, precision, F1 score, and accuracy. Further details regarding the performance evaluation are summarized in Appendix E1. Wilcoxon signed-rank test was employed to compare the ordered score feedback of “Preference”, as well as “difficulty of extracting key information”, while a paired *t*-test was used to compare the continuous data feedback labeled “Time spent”. In this study, *p* ≤ 0.05 is used to indicate statistical significance. The *p*-value reflects whether the responses of the report are different, assuming the null hypothesis means there is no difference between the reports. All statistical analysis and graphical results were computed using Python 3.9 with packages of Pandas, Numpy, Scipy, pingouin, and seaborn.

## Results

### OA categorization of the report collection

Among the 160 original reports included, 39.38% were categorized as no OA (63 of 160, K–L = 0), 28.13% as mild (45 of 160, K–L = 1, 2), 15.63% as moderate (25 of 160, K–L = 3), and 16.88% as severe (27 of 160, K–L = 4) based on the reference standard review (Fig. 1).

### Report creation performance

**Fig. 3:**
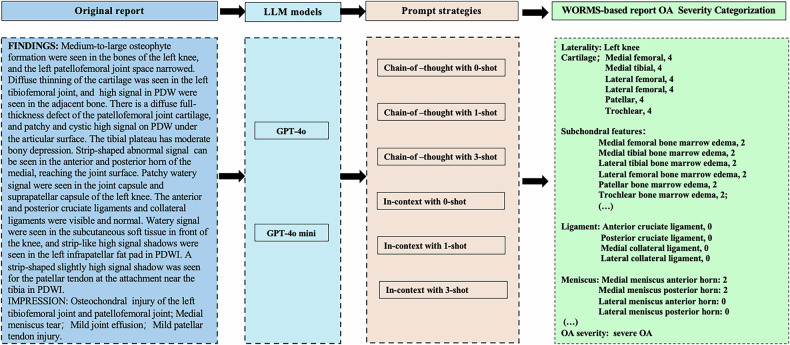
One exemplar case shows prompting strategies for models tasked with categorizing features and generating whole-organ MRI reports from original free-text reports, and further classifying OA severity. The provided template included fifty-one features and classification criteria for each. Two prompt strategies were used (COT and in-context). Moreover, three example reports with expected responses were included (0-shot, 1-shot, and 3-shot learning). All six strategies were applied to each model (GPT-4o and GPT-4o mini); thus, there is a total of 12 models. Identical prompts were used for both GPT-4o and GPT-4o mini. OA, osteoarthritis

**Fig. 4:**
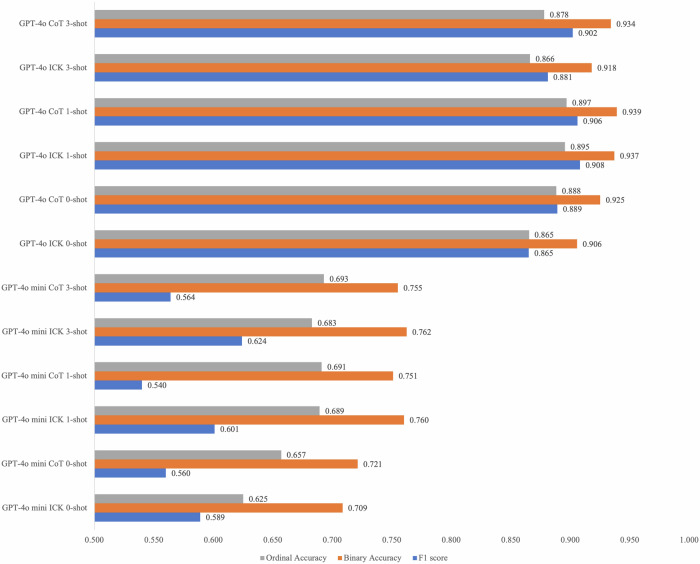
Performance of GPT-4o and GPT-4o mini with Different Prompts for Generating Whole-organ MRI Reports of the Knee from Original Free-text Reports. COT, chain-of-thought; ICK, in-context knowledge

Figure 3 illustrates one exemplary case, with LLM-generated reports and OA severity categorization prediction. The accuracy and F1 score of GPT-4o and GPT-4o mini with varying prompts for generating WORMS-based reports from original reports are presented in Fig. 4. Specifically, the GPT-4o–based model demonstrated significantly higher extraction F1 scores compared with GPT-4o mini (0.865–0.908 vs 0.540–0.624, Fig. 4). Similarly, GPT-4o exceeded GPT-4o mini in binary accuracy (90.6–93.9% vs 70.9–76.2%, *p* < 0.001) and ordinal accuracy (86.5–89.7% vs 62.5–69.3%, *p* < 0.001). Regarding different prompts and shot numbers, GPT-4o and GPT-4o mini exhibited part of similar and part of different trend. For GPT-4o mini, accuracy improved with an increasing number of shots, where 3-shot prompt yielded the highest accuracy (76.2% for binary accuracy, and 69.3% for ordinary accuracy, Fig. 4). While the trend for GPT-4o was slightly different: COT with 1-shot achieved the highest accuracy (93.9% for binary accuracy, and 89.7% for ordinal accuracy), while 0-shot and 3-shot prompts showed similar accuracy (Fig. 4).

### Performance of individual feature classification

The number of positive samples and performance metrics for individual features of the best setting for each model (COT with 1-shot for GPT-4o and COT with 3-shot for GPT-4o mini) are presented in Table 2. Overall, GPT-4o achieved higher recall, identifying more positive findings and thereby avoiding the omission of key information (87.3% s 46.7%, *p* < 0.001, Table 2), while maintaining higher precision compared to GPT-4o mini (94.2% vs 71.2%, *p* < 0.001, Table 2). GPT-4o demonstrated good to excellent accuracy in the categorization of individual features, with binary accuracy ranging from 82.5 to 100% and ordinal accuracy ranging from 76.2 to 100% (Table 2). Notably, GPT-4o outperformed GPT-4o mini in most features in terms of accuracy (*p* < 0.001, Table 2), except for a few features that had very low positive sample counts, including posterior cruciate ligament (28 samples, 17.5% positive) and several others with fewer than 20 positive samples (*p* > 0.05, Table 2).

**Table 2:** Performance for best settings of GPT-4o and GPT 4o mini for overall and individual features

	Positive Sample#	Recall			Precision			F1-score			Binary accuracy			Ordinal accuracy				Specificity	
		mini	4o	*p*	mini	4o	*p*	mini	4o	*p*	mini	4o	*p*	mini	4o	*p*	mini	4o	*p*
Overall	2739 (33.57%)	0.467	0.873	< 0.001	0.712	0.942	< 0.001	0.564	0.906	< 0.001	0.755	0.939	< 0.001	0.693	0.897	< 0.001	0.903	0.972	< 0.001
ACL	91 (56.88%)	0.692	0.956	< 0.001	1.0	1.0	> 0.99	0.818	0.978	> 0.99	0.825	0.975	< 0.001	0.669	0.9	< 0.001	1	1	> 0.99
PCL	28 (17.5%)	0.429	0.964	< 0.001	1.0	0.931	0.892	0.600	0.947	< 0.001	0.9	0.981	0.005	0.875	0.938	0.084	1	0.985	0.5
MCL	29 (18.13%)	0.655	0.966	0.007	0.950	0.903	0.942	0.775	0.933	0.007	0.931	0.975	0.113	0.9	0.962	0.047	0.992	0.977	0.625
LCL	29 (18.13%)	0.069	0.931	< 0.001	1.0	1.0	> 0.99	0.129	0.964	> 0.99	0.831	0.988	< 0.001	0.831	0.975	< 0.001	1	1	> 0.99
MF_Cartilage	97 (60.63%)	1	0.990	> 0.99	0.638	0.941	< 0.001	0.779	0.965	< 0.001	0.656	0.956	< 0.001	0.369	0.881	< 0.001	0.127	0.905	< 0.001
MF_BME	73 (45.63%)	1	0.932	0.069	0.480	0.919	< 0.001	0.649	0.925	< 0.001	0.506	0.931	< 0.001	0.388	0.85	< 0.001	0.092	0.931	< 0.001
MF_Subarticular cyst	24 (15%)	0.792	0.750	> 0.99	0.413	0.900	< 0.001	0.543	0.818	< 0.001	0.8	0.95	< 0.001	0.769	0.925	< 0.001	0.801	0.985	< 0.001
MF_Subarticular bone attrition	1 (0.625%)	0	0	> 0.99	0	0.0	> 0.99	0.0	0.0	> 0.99	0.906	0.975	0.018	0.906	0.975	0.018	0.912	0.981	0.013
MF_Marginal osteophyte	87 (54.38%)	0.989	0.931	0.123	0.705	0.942	< 0.001	0.823	0.936	< 0.001	0.769	0.931	< 0.001	0.481	0.812	< 0.001	0.507	0.932	< 0.001
MT_Cartilage	96 (60%)	0.573	0.948	< 0.001	0.809	0.938	0.021	0.671	0.943	< 0.001	0.662	0.931	< 0.001	0.488	0.869	< 0.001	0.797	0.906	0.118
MT_BME	70 (43.75%)	0.457	0.843	< 0.001	0.667	0.881	0.011	0.542	0.862	< 0.001	0.662	0.881	< 0.001	0.594	0.812	< 0.001	0.822	0.911	0.096
MT_Subarticular cyst	24 (15%)	0.25	0.750	0.001	0.600	0.947	0.066	0.353	0.837	0.001	0.863	0.956	0.006	0.844	0.931	0.021	0.971	0.993	0.375
MT_Subarticular bone attrition	22 (13.75%)	0	0.818	< 0.001	0	1.0	0.001	0.0	0.9	< 0.001	0.85	0.975	< 0.001	0.85	0.969	< 0.001	0.986	1	0.5
MT_Marginal osteophyte	91 (56.88%)	0.319	0.813	< 0.001	0.853	0.987	0.017	0.464	0.892	< 0.001	0.581	0.887	<0.001	0.488	0.781	< 0.001	0.928	0.986	0.219
LF_Cartilage	81 (50.63%)	0.58	0.889	< 0.001	0.618	0.911	< 0.001	0.598	0.9	< 0.001	0.606	0.9	< 0.001	0.494	0.85	< 0.001	0.633	0.911	< 0.001
LF_BME	58 (36.25%)	0.603	0.741	0.166	0.455	0.878	< 0.001	0.519	0.804	< 0.001	0.594	0.869	< 0.001	0.531	0.825	< 0.001	0.588	0.941	< 0.001
LF_Subarticular Cyst	20 (12.5%)	0.5	0.650	0.522	0.357	0.812	0.009	0.417	0.722	0.015	0.825	0.938	0.003	0.8	0.919	0.004	0.871	0.979	< 0.001
LF_Subarticular bone attrition	0 (0%)	n/a	n/a	n/a	n/a	n/a	n/a	n/a	n/a	n/a	0.975	0.988	0.680	0.975	0.988	0.680	0.975	0.988	0.688
LF_Marginal osteophyte	73 (45.63%)	0.973	0.822	0.006	0.732	0.923	0.005	0.835	0.87	< 0.001	0.825	0.887	0.152	0.612	0.812	< 0.001	0.701	0.943	< 0.001
LT_Cartilage	77 (48.13%)	0.364	0.805	< 0.001	0.778	0.861	0.411	0.496	0.832	< 0.001	0.644	0.844	< 0.001	0.569	0.794	< 0.001	0.904	0.88	0.791
LT_BME	54 (33.75%)	0.389	0.704	0.002	0.488	0.792	0.005	0.433	0.745	< 0.001	0.656	0.838	< 0.001	0.625	0.788	0.002	0.792	0.906	0.017
LT_Subarticular cyst	20 (12.5%)	0.3	0.600	0.112	0.429	0.923	0.021	0.353	0.727	0.016	0.863	0.944	0.023	0.85	0.919	0.080	0.943	0.993	0.039
LT_Subarticular bone attrition	9 (5.625%)	0	0.667	0.012	0	0.500	0.111	0.0	0.572	0.011	0.906	0.944	0.289	0.906	0.944	0.289	0.96	0.96	> 0.99
LT_Marginal osteophyte	73 (45.63%)	0.356	0.767	< 0.001	0.788	0.949	0.042	0.490	0.848	< 0.001	0.662	0.875	< 0.001	0.562	0.794	< 0.001	0.92	0.966	0.289
Patella_Cartilage	132 (82.50%)	0.614	0.955	< 0.001	1.0	1.0	> 0.99	0.761	0.977	> 0.99	0.681	0.963	< 0.001	0.394	0.881	< 0.001	1	1	> 0.99
Patella_BME	107 (66.88%)	0.673	0.879	< 0.001	0.783	0.989	< 0.001	0.724	0.931	< 0.001	0.656	0.912	< 0.001	0.5	0.775	< 0.001	0.623	0.981	< 0.001
Patella_Subarticular cyst	48 (30%)	0.104	0.937	< 0.001	0.625	0.957	0.018	0.178	0.947	< 0.001	0.713	0.969	< 0.001	0.706	0.894	< 0.001	0.973	0.982	> 0.99
Patella_Subarticular bone attrition	2(1.250%)	0	1.0	0.317	0	0.667	> 0.99	0.0	0.8	0.135	0.981	0.994	0.615	0.981	0.981	> 0.99	0.994	0.994	> 0.99
Patella_Marginal osteophyte	82 (51.25%)	0.585	0.768	0.019	0.842	0.969	0.033	0.690	0.857	0.005	0.731	0.869	0.003	0.556	0.806	< 0.001	0.885	0.974	0.039
Trochlea_Cartilage	106 (66.25%)	0.142	0.868	< 0.001	0.882	0.989	0.093	0.245	0.925	< 0.001	0.419	0.906	< 0.001	0.362	0.85	< 0.001	0.963	0.981	> 0.99
Trochlea_BME	88 (55%)	0.239	0.830	< 0.001	0.840	0.961	0.109	0.372	0.891	< 0.001	0.556	0.887	< 0.001	0.481	0.8	< 0.001	0.944	0.958	> 0.99
Trochlea_Subarticular cyst	36 (22.5%)	0.083	0.889	< 0.001	1.0	0.865	> 0.99	0.153	0.877	< 0.001	0.794	0.944	< 0.001	0.788	0.881	0.035	1	0.96	0.063
Trochlea_Subarticular bone attrition	1 (0.625%)	0	1.0	> 0.99	0	1.0	> 0.99	0.0	1.0	0.223	0.988	1.0	0.478	0.981	0.988	> 0.99	0.994	1	> 0.99
Trochlea_Marginal osteophyte	78 (48.75%)	0.179	0.692	< 0.001	0.824	0.947	0.256	0.294	0.800	< 0.001	0.581	0.831	< 0.001	0.562	0.762	< 0.001	0.963	0.963	> 0.99
MMAH	68 (42.5%)	0.044	0.794	< 0.001	1.0	0.900	> 0.99	0.084	0.844	< 0.001	0.594	0.875	< 0.001	0.594	0.819	< 0.001	1	0.935	0.031
MMPH	139 (86.88%)	0.151	0.878	< 0.001	1.0	1.0	> 0.99	0.262	0.935	> 0.99	0.263	0.894	< 0.001	0.188	0.781	< 0.001	1	1	> 0.99
LMAH	114 (71.25%)	0.096	0.798	< 0.001	0.846	0.948	0.422	0.172	0.867	< 0.001	0.344	0.825	< 0.001	0.306	0.781	< 0.001	0.957	0.891	0.453
LMPH	91 (56.88%)	0.231	0.857	< 0.001	0.808	0.918	0.223	0.359	0.886	< 0.001	0.531	0.875	< 0.001	0.475	0.8	< 0.001	0.928	0.899	0.754
Laterality	Left: Right—75:85	0.012	1	< 0.001	1.0	1.0	> 0.99	0.024	1.0	> 0.99	0.475	1.0	< 0.001	0.475	1.0	< 0.001	1	1	> 0.99

Three significant digits after the decimal point were kept

*ACL* anterior cruciate ligament, *PCL* posterior cruciate ligament, *MCL* medial collateral ligament, *LCL* lateral collateral ligament, *MF* medial femoral, *MT* medial tibial, *LF* lateral femoral, *LT* lateral tibial, *BME* bone marrow edema, *MMAH* medial meniscus anterior horn, *MMPH* medial meniscus posterior horn, *LMAH* lateral meniscus anterior horn, *LMPH* lateral meniscus posterior horn

^#^ Data are numbers of the reports, and data in parentheses are percentages

Table 3 adds more details on the binary accuracy performance across all 12 prompt strategies. GPT-4o achieved perfect accuracy on laterality selection across all prompts, significantly outperforming GPT-4o mini (100% vs 45.9–47.5%, *p* < 0.001). Both models exhibited excellent accuracy in four features (anterior cruciate ligament, posterior cruciate ligament, medial collateral ligament, and lateral collateral ligament), with accuracies exceeding 80%. For the remaining 34 features, GPT-4o demonstrated fair to excellent accuracy, ranging from 78.7 to 100% (Table 3), which was significantly higher than those of GPT-4o mini (13.7–98.7%, *p* < 0.001). When looking at different features, significantly higher accuracy in ligaments classification (with an average accuracy of 95.4% for GPT-4o and 83.1% for GPT-4o mini), while lower accuracy in cartilage, bone marrow edema, osteophyte, and meniscus were observed within the same models, no matter what prompt was used (*p* < 0.001, Fig. S1).

**Table 3:** Binary accuracy of GPT-4o and GPT 4o mini with different prompt strategies for individual feature

	GPT-4o mini 0-shot		GPT-4o mini 1-shot		GPT-4o mini 3-shot		GPT-4o 0-shot		GPT-4o 1-shot		GPT-4o 3-shot	
	ICK	CoT	ICK	CoT	ICK	CoT	ICK	CoT	ICK	CoT	ICK	CoT
ACL	0.969	0.969	0.818	0.748	0.819	0.825	0.994	0.987	0.994	0.975	0.987	0.987
PCL	0.887	0.9	0.887	0.906	0.894	0.9	0.969	0.981	0.969	0.981	0.975	0.962
MCL	0.887	0.906	0.881	0.906	0.95	0.931	0.981	1	0.981	0.975	0.975	0.981
LCL	0.95	0.931	0.874	0.843	0.85	0.831	0.969	0.975	0.987	0.987	0.994	0.987
MF_Cartilage	0.706	0.737	0.66	0.654	0.644	0.656	0.937	0.95	0.918	0.956	0.906	0.925
MF_BME	0.562	0.612	0.597	0.56	0.531	0.506	0.925	0.912	0.912	0.931	0.875	0.9
MF_Subarticular cyst	0.85	0.837	0.811	0.836	0.812	0.8	0.887	0.937	0.969	0.95	0.925	0.95
MF_Subarticular bone attrition	0.369	0.644	0.899	0.906	0.912	0.906	0.887	0.95	0.969	0.975	0.962	0.975
MF_Marginal osteophyte	0.706	0.719	0.786	0.774	0.75	0.769	0.894	0.912	0.912	0.931	0.894	0.95
MT_Cartilage	0.725	0.625	0.774	0.629	0.669	0.662	0.912	0.906	0.912	0.931	0.894	0.906
MT_BME	0.594	0.631	0.723	0.654	0.731	0.662	0.881	0.85	0.893	0.881	0.862	0.869
MT_Subarticular cyst	0.869	0.85	0.862	0.862	0.875	0.862	0.887	0.925	0.981	0.956	0.9	0.937
MT_Subarticular bone attrition	0.525	0.681	0.874	0.862	0.856	0.85	0.937	0.95	0.943	0.975	0.956	0.962
MT_Marginal osteophyte	0.712	0.675	0.673	0.591	0.675	0.581	0.875	0.844	0.912	0.887	0.9	0.969
LF_Cartilage	0.562	0.575	0.585	0.579	0.556	0.606	0.825	0.925	0.881	0.9	0.862	0.906
LF_BME	0.419	0.431	0.516	0.585	0.512	0.594	0.8	0.837	0.912	0.869	0.844	0.844
LF_Subarticular cyst	0.869	0.812	0.855	0.893	0.756	0.825	0.8	0.9	0.987	0.937	0.944	0.912
LF_Subarticular bone attrition	0.3	0.481	0.849	0.95	0.85	0.975	0.906	0.95	0.981	0.987	0.969	0.994
LF_Marginal osteophyte	0.656	0.694	0.73	0.774	0.681	0.825	0.887	0.869	0.887	0.887	0.869	0.931
LT_Cartilage	0.694	0.65	0.698	0.667	0.675	0.644	0.787	0.887	0.836	0.844	0.781	0.794
LT_BME	0.475	0.556	0.648	0.61	0.712	0.656	0.825	0.787	0.918	0.837	0.8	0.837
LT_Subarticular cyst	0.906	0.862	0.893	0.899	0.85	0.862	0.844	0.912	0.969	0.944	0.944	0.912
LT_Subarticular bone attrition	0.487	0.694	0.862	0.912	0.862	0.906	0.894	0.887	0.931	0.944	0.944	0.937
LT_Marginal osteophyte	0.631	0.631	0.679	0.648	0.681	0.662	0.844	0.819	0.843	0.875	0.844	0.919
Patella_Cartilage	0.644	0.7	0.711	0.66	0.862	0.681	0.969	0.994	0.969	0.962	0.956	0.975
Patella_BME	0.712	0.75	0.742	0.679	0.762	0.656	0.869	0.919	0.925	0.912	0.856	0.881
Patella_Subarticular cyst	0.756	0.75	0.761	0.717	0.794	0.712	0.919	0.956	0.962	0.969	0.95	0.925
Patella_Subarticular bone attrition	0.631	0.744	0.937	0.95	0.919	0.981	0.937	0.95	0.987	0.994	0.981	1
Patella_Marginal osteophyte	0.681	0.669	0.66	0.654	0.744	0.731	0.881	0.856	0.862	0.869	0.837	0.875
Trochlea_Cartilage	0.694	0.581	0.61	0.428	0.587	0.419	0.906	0.975	0.906	0.906	0.894	0.95
Trochlea_BME	0.675	0.606	0.61	0.553	0.606	0.556	0.869	0.906	0.918	0.887	0.894	0.887
Trochlea_Subarticular cyst	0.812	0.812	0.805	0.78	0.794	0.794	0.937	0.95	0.969	0.944	0.962	0.937
Trochlea_Subarticular bone attrition	0.662	0.781	0.962	0.969	0.95	0.987	0.931	0.962	0.981	1	0.969	0.994
Trochlea_Marginal osteophyte	0.637	0.65	0.616	0.522	0.656	0.581	0.819	0.794	0.811	0.831	0.806	0.869
MMAH	0.575	0.575	0.572	0.572	0.587	0.594	0.9	0.912	0.881	0.875	0.825	0.819
MMPH	0.162	0.137	0.201	0.17	0.337	0.262	0.869	0.856	0.881	0.894	0.869	0.919
LMAH	0.287	0.287	0.296	0.296	0.394	0.344	0.887	0.894	0.925	0.825	0.856	0.819
LMPH	0.506	0.462	0.472	0.44	0.512	0.531	0.837	0.856	0.893	0.875	0.819	0.844
Laterality	0.462	0.475	0.459	0.465	0.469	0.475	1	1	1	1	1	1

Three significant digits after the decimal point were kept

*ACL* anterior cruciate ligament, *PCL* posterior cruciate ligament, *MCL* medial collateral ligament, *LCL* lateral collateral ligament, *MF* medial femoral, *MT* medial tibial, *LF* lateral femoral, *LT* lateral tibial, *BME* bone marrow edema, *MMAH* medial meniscus anterior horn, *MMPH* medial meniscus posterior horn, *LMAH* lateral meniscus anterior horn, *LMPH* lateral meniscus posterior horn

### OA severity categorization

The accuracies of the models using different prompts for categorizing OA severity are illustrated in Table 4. GPT-4o with COT 1-shot prompt achieved the highest accuracy among the twelve prompt strategies, attaining 98.1% for binary and 96.2% for ordinary categorization. For each prompting strategy, GPT-4o was significantly more accurate than GPT-4o mini, both in the ICK (*p* < 0.001) and COT (*p* < 0.001) settings. Moreover, COT outperformed ICK (best ordinal accuracy: 96.2% vs 66.7% for GPT-4o; 47.5% vs 22.5% for GPT-4o mini; all *p* < 0.001). Within the COT framework, GPT-4o demonstrated the highest accuracy with the 1-shot prompt and the lowest accuracy with the 3-shot prompt (ordinal accuracy: 96.2% vs 89.4%, *p* = 0.03, Table 4); In contrast, GPT-4o mini exhibited a trend of increasing ordinal accuracy with a higher number of shots, although the differences were not statistically significant (*p* = 0.07). Notably, all three GPT-4o COT models surpassed the accuracy threshold established by orthopedic surgeons, whereas the ICK prompt did not meet the standard (Table 4).

**Table 4:** The accuracy of OA severity categorization for GPT-4o and GPT-4o mini of different prompts

	Binary accuracy (%)	Ordinal accuracy (%)
GPT-4o mini ICK 0-shot	69.4	19.4
GPT-4o mini CoT 0-shot	65.6	36.9
GPT-4o mini ICK 1-shot	70.4	24.5
GPT-4o mini CoT 1-shot	78.6	45.3
GPT-4o mini ICK 3-shot	63.7	22.5
GPT-4o mini CoT 3-shot	**68.7**	**47.5**
GPT-4o ICK 0-shot	84.4	68.7
GPT-4o CoT 0-shot	96.9	92.5
GPT-4o ICK 1-shot	86.8	66.7
GPT-4o CoT 1-shot	**98.1**	**96.2**
GPT-4o ICK 3-shot	84.4	62.5
GPT-4o CoT 3-shot	97.5	89.4

The accuracy of the best setting is presented in bold

**Fig. 5:**
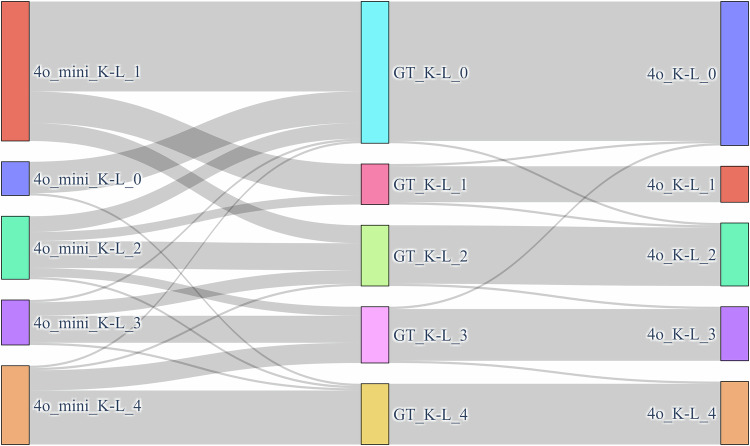
The Sankey diagram of performance for GPT-4o COT 1-shot and GPT 4o mini COT 3-shot across different K–L grades. The left column represents the GPT-4o mini, the middle column represents the ground truth of K–L grades, and the right column represents GPT-4o. GPT-4o demonstrates better performance than GPT-4o mini. Both prompts illustrate their best performance in K–L grade 4

We further examined the performance of different prompts across different K–L grades. GPT-4o achieved the highest performance with the COT 1-shot prompt (Cohen's kappa value = 0.95). While for GPT-4o mini, the best performance was observed with the COT 3-shot prompt (Cohen's kappa value = 0.371). Figure 5 gave the detailed performance of these two prompts. Notably, both prompts demonstrated their best performance (recall = 1 for GPT-4o and recall = 0.889 for GPT-4o mini) in K–L grade 4.

### Orthopedic surgeon review

**Fig. 6:**
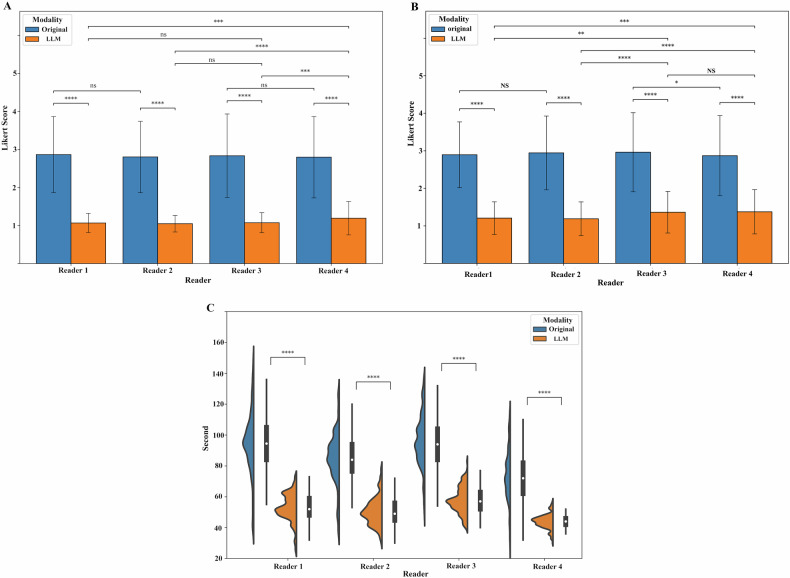
Orthopedic surgeon review results. **A** Preference for each report based on a 5-point Likert scale, where higher scores represented less preference; **B** degree of difficulty in extracting key information based on a 5-point Likert scale, where higher scores represented greater difficulty; and (**C**) the amount and of time spent in seconds for each report. LLM, large language model

An overview of orthopedic surgeon review results is displayed in Fig. 6. All four orthopedic surgeons preferred LLM-generated reports over original reports (Fig. 6A, *p* < 0.001), with 99.53% vs 35.94% of reports being scaled as “like” or “very like” and 0% vs 21.09% reports being rated as “unlike” or “very unlike”, respectively. The orthopedic surgeons also found it significantly easier to extract key information from LLM-generated reports compared to the original reports (*p* < 0.001, Fig. 6B), with 96.72% vs 29.22% of reports scored as “easy” or “very easy”, and 0% vs 21.56% scored as “difficult” or “very difficult”, respectively. Furthermore, surgeons were more efficient in extracting features using LLM-generated reports (Fig. 6C), spending an average of 58.65% less time on LLM-generated reports compared with original reports (51.27 ± 9.41 s vs 87.42 ± 20.26 s, *p* < 0.001).

## Discussion

The potential of LLMs to flexibly transform free-text reports into structured ones has been reported in a few studies [17–19]. However, research focuses on the generation of whole-organ, multi-feature knee MRI reports, and the prediction of OA severities remains limited. The purpose of this study was to evaluate the performance of LLMs in automatically generating WORMS-based structural reports from original reports and to explore their ability to classify knee OA severity using various prompting strategies.

To enhance the performance of LLMs in our complex classification tasks, we explored six prompting strategies across two LLM models: ICK and COT, utilizing 0, 1, and 3-shot approaches, respectively. Among these strategies, GPT-4o COT with 1-shot prompt demonstrated the best performance in both report generation and OA severity prediction tasks, achieving an accuracy of 93.9% and 98.1%, respectively. These results significantly exceed the previously established accuracy threshold of 85% for preliminary categorization of LLM models [10], as well as previously published data (70% accuracy) [13]. This makes it an exciting application in knee OA care, automating the generation of accurate classifications of features and OA severity tasks that are traditionally time-consuming for radiologists. We further explored their performance on specific K–L classification. Notably, K–L = 4 (the most severe classification of OA) showed the best performance in both models. This result is reasonable, as the most severe classification represents the most easily identifiable characteristic features. When comparing different prompts, COT outperformed ICK prompts for both models, aligning with findings from earlier studies [20, 21]. Within the COT prompt, GPT-4o with 1-shot performed better than 3-shot, while the GPT-4o mini improved with an increased number of shots. It is suspected that the COT for GPT-4o may be sufficiently robust that additional shots (like 3-shot) could hinder its performance, while GPT-4o mini may require more shots to enhance its effectiveness [16].

In our study, four orthopedic surgeons evaluated two types of reports based on their preferences, the degree of difficulty of extracting key information, and the time spent. These three aspects have been previously identified as crucial for assessing the quality of LLM-generated reports [10, 22]. Our findings revealed that the surgeons strongly preferred structured WORMS-based reports produced by LLMs when having access to both original and LLM-generated reports in a real-world clinical setting. This positive feedback aligns with findings from prior studies [9, 10]. Moreover, the challenge of analyzing large volumes of free-text Radiology reports and summarizing key information imposes a substantial burden on clinicians, potentially leading to misinformation, inappropriate care, and adverse health outcomes in practice. The high efficiency and ease of use scored by the surgeons in the present study suggest that integrating LLMs into clinical OA workflows could greatly enhance productivity and alleviate the documentation burden faced by clinicians [23].

Although GPT-4o demonstrated proficiency in generating WORMS-based knee MRI reports, it still made several clinically significant errors, primarily due to misinterpretations in the classification of individual features, with GPT-4o mini exhibiting higher incidences of such errors. One common limitation shared by both models was their relatively weaker performance in cartilage classification compared to other features (Fig. S1). This limitation is largely attributed to the complexities arising from the multi-location and multi-classification nature of the cartilage feature. Specifically, there are six subregions of cartilage, each with eight classifications (as detailed in Appendix E1), resulting in a total of 48 distinct classifications for cartilage features alone. Our finding reveals that longer text with numerous features increases the likelihood of LLMs making factual inconsistencies and generating misleading summaries. This finding aligns with the work of Liyan Tang et al [9]. In the future, we plan to collect additional data to fine-tune these LLMs, aiming to enhance their understanding of specialized terminology used in the WORMS classification. Although these models have not yet achieved the accuracy required to replace expert healthcare professionals [24], our results highlight their potential as valuable tools in supervised settings, where they can help alleviate the burden on radiologists handling complex and specialized clinical tasks.

There were several limitations to our study. First, we relied on original reports, which occasionally contained ambiguous descriptions or conveyed uncertainty, introducing a degree of subjectivity in their interpretation. Second, the models were not externally validated on reports from other institutions. But they were also not fine-tuned on internal reports, with only a few examples provided for training. Additionally, the evaluation was conducted on reports from different radiologists, which may have introduced variability due to individual reporting styles. Further research should prioritize external validation to account for institutional variations in reporting styles and contents.

In conclusion, GPT-4o excelled at generating WORMS-based knee MRI reports from original free-text reports and categorizing OA severity. Surgeons found LLM-generated reports to be more efficient for extracting key information and preferred them over original reports. Future research should investigate the efficacy of LLMs in summarizing reviews that involve longer contexts and multiple modalities. Additionally, integrating LLMs with other AI technologies, such as vision-language models, could further enhance their capabilities in clinical applications.

## Supplementary information

ELECTRONIC SUPPLEMENTARY MATERIAL

## Data Availability

The data are available from the corresponding author upon reasonable request.

## Abbreviations

COT: Chain-of-thought
ICK: In-context knowledge
K–L: Kellgren–Lawrence
LLM: Large language model
OA: Osteoarthritis
WORMS: Whole-organ MRI score
