# Supplementary material for: Large language models for efficient whole-organ MRI score-based reports and categorization in knee osteoarthritis
Source: Insights Imaging. 2025 May 14;16:100. doi: 10.1186/s13244-025-01976-w (PMC12078906; doi:10.1186/s13244-025-01976-w)
Supplement: Supplementary file 1 — ELECTRONIC SUPPLEMENTARY MATERIAL [file 13244_2025_1976_MOESM1_ESM.docx]

**Appendix S1**

**Materials and Methods**

**The classification criteria for each feature basing on WORMS classification(1) and was modified.**

1. **Cartilage and subchondral features**

**Subdivision**

**The femoral cartilage** was divided into medial (MF) and lateral (LF) condyles. The boundary between MF and LF was defined by a plane aligned with the lateral wall of the femoral notch. The subchondral component of femoral region extended perpendicularly from the articular surface to the level of an imaginary line connecting the anterior and posterior osteochondral junctions.

**The tibial cartilage** was divided into medial tibial plateau (MT) and lateral tibial plateau (LT). The subchondral component of tibial region extended 2 cm beneath the articular surface.

**The patellar and trochlear cartilage** were not further divided. The subchondral component of patellar region extended the full thickness of the bone to the opposite cortex. The subchondral component of trochlear region extended perpendicularly from the articular surface to the level of an imaginary line connecting the anterior and posterior osteochondral junctions.

**Cartilage classification:** Cartilage signal and morphology was scored in each of the articular-surface regions using an eight-point scale 0=normal thickness and signal; 1=normal thickness but increased signal on T2-weighted images; 2.0=partial-thickness focal defect <1 cm in greatest width; 2.5=full- thickness focal defect <1 cm in greatest width; 3=multiple areas of partial-thickness (Grade 2.0) defects intermixed with areas of normal thickness, or a Grade 2.0 defect wider than 1 cm but <75% of the region; 4=diffuse (≥75% of the region) partial-thickness loss; 5=multiple areas of full- thickness loss (grade 2.5) or a grade 2.5 lesion wider than 1 cm but <75% of the region; 6=diffuse (≥75% of the region) full-thickness loss.

**Subchondral features classification:**

- Subarticular bone marrow abnormality was defined as poorly marginated areas of increased signal intensity in the normally fatty epiphyseal marrow on fat-suppressed T2-weighted images. This feature was graded in each of the 14 articular surface regions from 0 to 3 based on the extent of regional involvement: 0=none; 1=<25% of the region/little; 2=25% to 50% of the region/moderate; 3=>50% of the region/large-scale.
- Subarticular cysts were identified as foci of markedly increased signal in the subarticular bone with sharply defined, rounded margins and no evidence of internal marrow tissue or trabecular bone. Bone cysts were graded in each region, from 0 to 3 based on the extent of regional involvement, as for bone marrow abnormality: 0=none; 1=<25% of the region/a little; 2=25% to 50% of the region/moderate; 3=>50% of the region/large-scale.
- Flattening or depression of the articular surfaces was termed bone attrition and graded from 0 to 3 based on the subjective degree of deviation from the normal contour: 0=normal; 1=mild; 2=moderate; 3=severe. flattening=Grade 1, slight concavity=Grade 2, and marked concavity=Grade 3.
- Osteophytes were graded from 0 to 7 using the following scale: 0=none; 1=equivocal; 2=small; 3=small-moderate; 4=moderate; 5=moderate-large; 6=large; 7=very large

2.**The anterior cruciate ligament (ACL), posterior cruciate ligament (PCL), medial collateral ligament (MCL), and lateral collateral ligament (LCL)** were independently scored based on a 5-point scale(2): 0=intact, 1=signal abnormality, 2=partial tear, and 3=full tear, and 4= reconstructed.

**3.The meniscus** was subdivided into anterior horn and posterior horn of the medial and lateral menisci and were graded separately from 0 to 4: 0=intact; 1=minor radial tear or parrot-beak tear; 2=non-displaced tear or prior surgical repair; 3=displaced tear or partial resection; 4=complete maceration/destruction or complete resection.

**4.Synovial thickening and joint effusion** were not distinguished from each other. So they were graded collectively from 0 to 3 in terms of the estimated maximal distention of the synovial cavity: 0=normal; 1=<33% of maximum potential distention; 2=33%–66% of maximum potential distention; 3=>66% of maximum potential distention.

Loose bodies in the synovial cavity were scored from 0 to 3 based on number: 0=none; 1=1 loose body; 2=2 loose bodies; 3=3 or more loose bodies.

**5.****Synovial cysts or bursal collections** about the knee were specified (e.g., popliteal, anserine, semimembranosus, meniscal, infrapatellar, prepatellar, tibiofibular, etc.) and graded 1 to 3 based subjectively on size: 1=small-scale, 2=medium-scale, and 3=large-scale.

All of the above features were classified ordinarily according to the modified WORMS classification and then were categorized binarily as score of 0 for negative and scores of bigger than 0 for positive.

**The details of** **Kellgren-Lawrence grading (K-L) system.**

The Kellgren-Lawrence (K-L)grading system, endorsed by the World Health Organization since 1961 as the gold standard for evaluating knee osteoarthritis (OA) severity through X-ray analysis (3). The X-ray was reviewed by 3 board-certified musculoskeletal radiologists to determine the K-L Classification for each patient. To determine a reference standard for the K-L classification for each case, images were independently reviewed by two radiologists (with 16 and 10 years of experience, respectively), with disagreements discussed with an advanced radiologist to reach consensus (with 38 years of experience). The K-L classification was established into five levels (0 to 4), with higher numbers indicating more severe damage: 0, normal knee; 1, suspected OA (suspicious joint space narrowing with possible osteophyte formation); 2, mild OA (possible joint space narrowing, definite osteophyte formation); 3, moderate OA (multiple osteophyte formations, definite joint space narrowing, subchondral sclerosis); and 4, severe OA (marked joint space narrowing, osteophytes, definite bone deformity, severe sclerosis).

**Details about the Prompting strategies.**

The extractable findings and classifications for each of the 39 key features, as well as the OA severity were explored based on different prompt strategies. Specifically, two prompting strategies, in-context knowledge and chain-of-thought were used with 0-shot, 1-shot and 3-shot learning was applied. The first strategy (“in-context”) explicitly provided the model with WORMS classification in the prompt, allowing it to reference the criteria while categorizing every feature. The second strategy involved breaking down WORMS classification and OA severity criteria more explicitly into manageable detailed pieces.

**Data Analysis**

Performance between GPT-4o and GPT-4o mini models was calculated as recall, precision, F1 score, and accuracy at different aspects. For overall accuracy comparison, a mixed-effects logistic regression model was also constructed to predict whether an extractable feature was correctly identified, with a random effect for reports and a fixed effect for methods. The random effect helped account for within-report correlation between the 39 features. When looking at the 39 features separately, by using Bonferroni correction, *P* values of less than .00128 were considered to indicate a significant difference. The accuracy results were compared between best settings of two LLM models with the McNemar test. Confidence interval (CI) was calculated based on Wald approximation method and Wilson score if the results close to the extremes or the sample size is small. The *P* value of the fixed effect was used to assess if there was a difference in accuracy.

1. Peterfy CG, Guermazi A, Zaim S, Tirman PF, Miaux Y, White D, Kothari M, Lu Y, Fye K, Zhao S, Genant HK. Whole-Organ Magnetic Resonance Imaging Score (WORMS) of the knee in osteoarthritis. Osteoarthritis Cartilage 2004;12(3):177-190. doi: 10.1016/j.joca.2003.11.003

2. Astuto B, Flament I, N KN, Shah R, Bharadwaj U, T ML, M DB, Pedoia V, Majumdar S. Automatic Deep Learning-assisted Detection and Grading of Abnormalities in Knee MRI Studies. Radiol Artif Intell 2021;3(3):e200165. doi: 10.1148/ryai.2021200165

3. Schiphof D, Boers M, Bierma-Zeinstra SM. Differences in descriptions of Kellgren and Lawrence grades of knee osteoarthritis. Ann Rheum Dis 2008;67(7):1034-1036. doi: 10.1136/ard.2007.079020
